# Metformin reduces new-onset atrial fibrillation risk rather than atrial fibrillation burden in type 2 diabetes patients: A case-control study

**DOI:** 10.1016/j.heliyon.2024.e30992

**Published:** 2024-05-09

**Authors:** Chongxia Zhong, Jian Bai, Xinhong Qu, Yihai Liu, Zhu Li, Han Hao, Shiyang Qiao, Zhe Zhang, Xiaoying Xu, Jiayi Si, Wei Xu, Biao Xu, Lina Kang

**Affiliations:** a Department of Cardiology, Nanjing Drum Tower Hospital, Affiliated Hospital of Medical School, Nanjing University, Nanjing, 210008, China; b Department of Cardiology, Nanjing Drum Tower Hospital, Clinical School of Nanjing Medical University, Nanjing, China; c Department of Cardiology, Nanjing Drum Tower Hospital, Clinical School of Nanjing University of Traditional Chinese Medicine, Nanjing, China; d Nanjing Key Laboratory for Cardiovascular Information and Health Engineering Medicine, China

**Keywords:** Diabetes mellitus, Metformin, New-onset atrial fibrillation, Atrial fibrillation burden, Pacemaker

## Abstract

**Background:**

The effects of metformin on atrial fibrillation (AF) in type 2 diabetes patients remain unclear. We aimed to explore the effects of metformin on AF, including new-onset AF and AF burden, in type 2 diabetes patients with pacemakers.

**Methods and results:**

This retrospective study included a total of 227 patients. Based on the presence of paroxysmal AF, the patients were divided into a paroxysmal AF group (n = 80) and a non-AF group (n = 147). In the non-AF group, a significant association was observed between metformin use and a lower risk of new-onset AF in multivariable Cox hazards models (hazard ratio [HR]: 0.36; 95 % confidence interval [CI]: 0.14–0.91; p = 0.0311*) when adjusted for age, sex, body mass index (BMI), drinking, smoking, left atrial dimension, creatinine, complications, and drugs. In the paroxysmal AF group, univariable analysis indicated no association between the AF burden and metformin use (p = 0.817). Furthermore, when adjusted for metformin use, age, sex, BMI, drinking, smoking, cardiovascular disease, myocardial infarction, heart failure, stroke, and ejection fraction in multivariable Cox hazards models, we found a lower proportion of major adverse cardiovascular events (MACEs) both in the total (HR: 0.28; 95 % CI: 0.1–0.82; p = 0.0202*) and the non-AF group (HR: 0.19; 95 % CI: 0.05–0.79; p = 0.0223*) compared to that in the AF group (HR: 0.31; 95 % CI: 0.02–4.41; p = 0.3879).

**Conclusion:**

In type 2 diabetes patients with pacemakers, metformin reduced the probability of new-onset AF instead of addressing the AF burden. Furthermore, metformin therapy decreased the incidence of MACEs in type 2 diabetes patients without AF.

## 1 Introduction

Atrial fibrillation (AF) is the most common sustained arrhythmia in clinical practice, resulting in increased morbidity such as stroke, heart failure (HF), and death, thus imposing a significant global health burden [1]. In 2019, the global burden of AF was estimated to be 59.7 million, and its prevalence is projected to more than double over the next three decades [2].

Diabetes mellitus (DM) is significantly associated with AF. Compared to that in diabetes patients without AF, those with AF have an increased risk of major coronary events, stroke, HF, cardiovascular death, and mortality [3]. The mechanisms of DM in AF include metabolic disorders and structural and electrical remodelling [4].

There is currently no preventive therapy for AF. Metformin is a classic first-line antidiabetic drug used worldwide, with a history of use beginning in the 1920s. In addition to regulating blood sugar levels, the pleiotropic effects of metformin on health have been explored, particularly its positive effects on aging-related morbidities, such as obesity, metabolic syndrome, cardiovascular disease, cancer, cognitive decline, and mortality [5]. Numerous studies have investigated the effects of metformin on AF in DM patients. In a retrospective study involving 645,710 DM patients, those who used metformin had a lower AF risk than those who did not use antidiabetic medication [6]. In a retrospective study aimed at investigating the risk of atrial and ventricular arrhythmia in diabetes patients taking different oral antidiabetic drugs, metformin lowered the risk of AF and ventricular arrhythmia compared with that of sulfonylureas [7]. Another retrospective observational study reported that type 2 DM patients treated with metformin had a lower risk of hospitalisation due to AF [8]. Nevertheless, contrary conclusions have been drawn in other studies. In a cohort study including 25,117 patients with either type 1 or 2 DM during a mean follow-up of 4.8 ± 3.5 years, metformin was associated with a higher risk of AF [9]. Compared with that of sulfonylureas, metformin is associated with a higher risk of incident AF, stroke, cardiovascular mortality, and all-cause mortality [10]. Furthermore, a meta-analysis found that metformin therapy increases the risk of AF/AFL in diabetes patients [11].

Previous studies exclusively relied on electrocardiograms for diagnosing AF, potentially missing paroxysmal asymptomatic AF and failing to assess AF burden. Given the conflicting conclusion in previous research, we conducted a study of metformin's impact on AF, including new-onset AF and AF burden in diabetes patients with pacemakers.

## 2 Materials and methods

### 2.1 Study participants

The study protocol was approved by the ethics committee of Nanjing Drum Tower Hospital. This single-centre retrospective study enrolled 227 diabetes patients with pacemaker implantation from the Department of Cardiology, Nanjing Drum Tower Hospital, from 2018 to 2022. The exclusion criteria were 1) type of diabetes other than type 2 diabetes, 2) diagnosed with persistent AF or receiving catheter ablation for AF, 3) accepting cardiac resynchronisation therapy (CRT/CRTD), 4) lack of data at admission, and 5) lost to follow-up.

### 2.2 Study protocol

Based on the presence or absence of paroxysmal AF, 227 patients were divided into paroxysmal AF and non-AF groups. The baseline diagnosis of paroxysmal AF was based on a 12-lead electrocardiogram, dynamic electrocardiogram, or electrocardiographic monitoring at admission. Baseline data included age, sex, drinking, smoking, physical examination (BMI and blood pressure), laboratory data (HbA1c, fasting blood glucose, creatinine [Cr], and C-reactive protein [CRP]), ultrasonic cardiogram (LAD and EF), combined disease (hypertension, HF, and MI), and medication (e.g., antidiabetic drugs, antihypertensive, and antiarrhythmic drugs). All baseline data were collected upon admission. For follow-up, patients were recalled to the pacemaker outpatient service at 1, 6, and 12 months after pacemaker implantation in the first year and at least once a year thereafter. The diagnosis of new-onset AF was based on pacemaker programming control, with major parameters including AT/AF time, AT/AF burden (%), AT/AF longest duration, and AT/AF total time. Data on major adverse cardiovascular events (MACEs) and whether patients continued to take or discontinued metformin were also collected from the outpatient department. MACEs were defined as revascularization, myocardial infarction, unstable angina, or cardiac death.

### 2.3 Statistical analysis

Continuous variables conforming to normal distribution are displayed as mean ± SD. Continuous variables that did not conform to a normal distribution are shown as medians with interquartile ranges (IQR). Categorical variables are expressed as numbers and percentages. Continuous and categorical variables were compared using the Student's t-test and χ^2^ test. The association between metformin therapy and new-onset AF or MACEs was assessed using Kaplan–Meier analysis and Cox proportional hazards regression. In the Cox proportional hazards regression analysis investigating the association between metformin and MACEs, Model 1 was adjusted for metformin use, age, and sex. Model 2 was further modified for BMI, alcohol consumption, and smoking status. Model 3 was adjusted for CVD, MI, HF, stroke, and EF. In the Cox proportional hazards regression analysis exploring the link between metformin and new-onset AF, Model 1 was adjusted for metformin use, age, and sex. Model 2 was further adjusted for BMI, alcohol consumption, and smoking status. Model 3 was further modified for LAD, Cr, complications, and medication. Statistical significance was defined as a two‐sided p < 0.05. All analyses were conducted using R software (R Statistics, version 4.1.3).

## 3 Results

### 3.1 Metformin decreased new-onset AF risk in type 2 diabetes patients

**Fig. 1:**
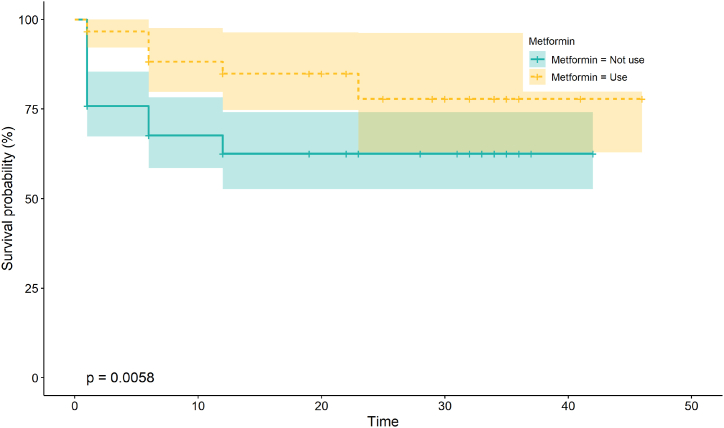
Kaplan–Meier curves comparing the probability of new-onset atrial fibrillation with and without metformin use. Patients who received metformin had a lower risk of new-onset AF (P < 0.05). The green line represents patients who did not use metformin, and the yellow line indicates patients using metformin. (For interpretation of the references to colour in this figure legend, the reader is referred to the Web version of this article.)

**Fig. 2:**
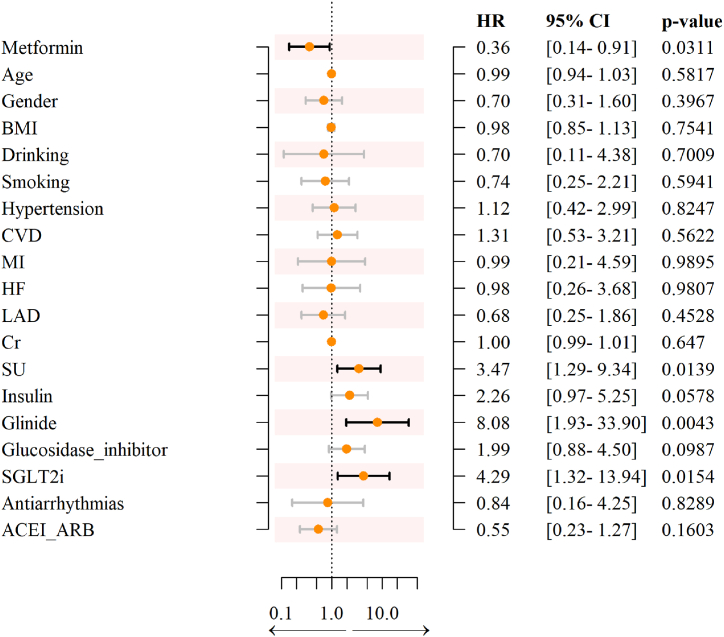
Risk of Metformin use on new-onset AF. Cox proportional hazards regression was used, and a forest plot was constructed. When adjusted for age, sex, BMI, alcohol consumption, smoking, LAD, Cr, complications, and drugs, metformin was identified as the independent protective factor for preventing new-onset AF in diabetes patients (hazard ratio [HR]: 0.36; ±95 % confidence interval [CI]: 0.14–0.91; p = 0.0311*). **ACEI**: angiotensin converting enzyme inhibitor; **ARB**: angiotensin receptor blocker; **Cr**: creatinine; **CVD**: cardiovascular disease; **HF**: heart failure; **HR**: hazard ratio; **LAD**: left atrial dimension; **MI**: myocardial infarction; **SGLT2i**: sodium-dependent glucose transporters 2 inhibitor; **SU**: sulfonylurea.

A total of 174 diabetes patients without paroxysmal AF were enrolled in our study. Patients on metformin were younger (p = 0.001***), consumed more alcohol, and exhibited better renal function than those in the non-metformin group (Table 1). The Kaplan–Meier curve indicated that patients taking metformin had a lower risk of developing new-onset atrial AF (Fig. 1). In univariable analysis, the use of metformin was associated with a significantly lower risk of AF, whereas the use of glinide had the opposite effect. In addition, advanced age, male sex, lower BMI, and a history of CVD, MI, and HF were risk factors for new-onset AF, although no statistical significance was observed (Table 2). In multivariable Cox hazards models, after adjusting for age, sex, BMI, alcohol consumption, smoking status, LAD, Cr, complications, and drugs, metformin was identified as the independent protective factor for preventing new-onset AF in diabetes patients (hazard ratio [HR]: 0.36; 95 % confidence interval [CI]: 0.14–0.91; p = 0.0311*) (Table 3 and Fig. 2).

**Table 1:** Clinical characteristics of DM patients without AF.

	Overall (n = 147)	Non-metformin (n = 87)	Metformin (n = 60)	p
Female sex (%)	60 (40.8)	33 (37.9)	27 (45.0)	0.4
Age (years)	75.00 [68.00, 81.00]	80.00 [69.00, 83.50]	71.00 [68.00, 77.00]	0.001***
BMI (kg/m^2^)	24.46 (3.11)	23.81 (3.02)	25.41 (3.01)	0.002**
Systolic pressure (mmHg)	130.31 (12.20)	130.16 (12.77)	130.52 (11.43)	0.863
Dystolic pressure (mmHg)	70.00 [65.00, 74.00]	70.00 [65.00, 75.50]	70.00 [66.00, 74.00]	0.657
Hypertension (%)	109 (74.1)	67 (77.0)	42 (70.0)	0.346
Hypercholesterol (%)	33 (22.4)	20 (23.0)	13 (21.7)	1
CVD (%)	37 (25.2)	26 (29.9)	11 (18.3)	0.126
MI (%)	8 (5.4)	7 (8.0)	1 (1.7)	0.142
HF (%)	15 (10.3)	12 (14.1)	3 (5.0)	0.099
Stroke (%)	24 (16.3)	14 (16.1)	10 (16.7)	1
CKD (%)	31 (21.2)	25 (29.1)	6 (10.0)	0.007**
Drinking (%)	16 (10.9)	4 (4.6)	12 (20.0)	0.006**
Smoking (%)	28 (19.0)	16 (18.4)	12 (20.0)	0.833
Cr (μmol/L)	70.00 [61.00, 87.00]	75.00 [64.50, 91.50]	65.00 [50.88, 77.50]	0.001***
CRP (mg/L)	3.60 [2.60, 5.00]	3.70 [2.80, 5.15]	3.10 [2.40, 4.60]	0.087
HbA1c (%)	7.10 [6.50, 8.30]	6.90 [6.50, 8.00]	7.10 [6.60, 8.67]	0.364
LAD (cm)	4.10 [3.92, 4.40]	4.10 [4.00, 4.40]	4.10 [3.84, 4.35]	0.474
EF (%)	58.00 [56.00, 60.00]	58.00 [55.50, 60.00]	59.00 [57.00, 60.00]	0.043*
Insulin (%)	40 (27.2)	30 (34.5)	10 (16.7)	0.023*
TZD (%)	1 (0.7)	0 (0.0)	1 (1.7)	0.408
SU (%)	27 (18.4)	12 (13.8)	15 (25.0)	0.128
Glinide (%)	6 (4.1)	5 (5.7)	1 (1.7)	0.401
Glucosidase inhibitor (%)	57 (38.8)	41 (47.1)	16 (26.7)	0.016*
GLP1 (%)	147 (100.0)	87 (100.0)	60 (100.0)	NA
DPP-4i (%)	32 (21.8)	17 (19.5)	15 (25.0)	0.542
SGLT2i (%)	13 (8.8)	6 (6.9)	7 (11.7)	0.381
Anticoagulant (%)	6 (4.1)	5 (5.7)	1 (1.7)	0.401
Antiplatelet (%)	67 (45.6)	46 (52.9)	21 (35.0)	0.043*
B-blocker (%)	47 (32.0)	29 (33.3)	18 (30.0)	0.721
Statins (%)	99 (67.3)	61 (70.1)	38 (63.3)	0.475
Anti-arrhythmia (%)	7 (4.8)	7 (8.0)	0 (0.0)	0.042*
ACEI/ARB (%)	65 (44.2)	39 (44.8)	26 (43.3)	0.868
Sacubatrovalsartan (%)	4 (2.7)	2 (2.3)	2 (3.3)	1
Aldactone (%)	15 (10.2)	11 (12.6)	4 (6.7)	0.28
Digoxin (%)	0 (0)	0 (0)	0 (0)	NA
CCB (%)	2 (1.4)	1 (1.1)	1 (1.7)	1
Furosemide (%)	14 (9.5)	12 (13.8)	2 (3.3)	0.044*

**AF:** atrial fibrillation; **ACEI**: angiotensin converting enzyme inhibitor; **ARB**: angiotensin receptor blocker; **CCB**: calcium channel blockers; **CKD**: chronic kidney disease; **Cr**: creatinine; **DM:** diabetes mellitus; **DPP-4i**: dipeptidyl peptidase 4 inhibitor; **GLP1**: glucagon-like peptide-1; **HF:** heart failure; **SGLT2i**: sodium-dependent glucose transporters 2 inhibitor; **SU**: sulfonylurea; **TZD**: thiazolidinediones.

**Table 2:** Univariable analysis of new-onset AF.

	Overall (n = 147)	No new-onset AF (n = 108)	New-onset AF (n = 39)	p
Female sex (%)	60 (40.8)	45 (41.7)	15 (38.5)	0.85
Age (year)	74.75 (9.46)	74.05 (9.16)	76.69 (10.11)	0.135
BMI (kg/m^2^)	24.46 (3.11)	24.72 (3.23)	23.67 (2.60)	0.08
Systolic pressure (mmHg)	130.31 (12.20)	130.70 (12.49)	129.21 (11.43)	0.513
Dystolic pressure (mmHg)	70.00 [65.00, 74.00]	70.00 [65.00, 74.25]	70.00 [64.50, 74.00]	0.456
Hypertension (%)	109 (74.1)	82 (75.9)	27 (69.2)	0.404
Hypercholesterol (%)	33 (22.4)	24 (22.2)	9 (23.1)	1
CVD (%)	37 (25.2)	25 (23.1)	12 (30.8)	0.391
MI (%)	8 (5.4)	5 (4.6)	3 (7.7)	0.438
HF (%)	15 (10.3)	10 (9.4)	5 (12.8)	0.548
Stroke (%)	24 (16.3)	20 (18.5)	4 (10.3)	0.315
Renal (%)	31 (21.2)	22 (20.6)	9 (23.1)	0.82
Drinking (%)	16 (10.9)	14 (13.0)	2 (5.1)	0.238
Smoking (%)	28 (19.0)	20 (18.5)	8 (20.5)	0.814
Cr (μmol/L)	70.00 [61.00, 87.00]	69.00 [59.75, 86.25]	76.00 [63.50, 87.00]	0.23
CRP (mg/L)	3.60 [2.60, 5.00]	3.50 [2.60, 5.10]	3.90 [2.60, 4.85]	0.613
HbA1c (%)	7.10 [6.50, 8.30]	7.10 [6.50, 8.30]	7.05 [6.65, 8.05]	0.92
LAD (cm)	4.10 [3.92, 4.40]	4.10 [4.00, 4.40]	4.10 [3.91, 4.31]	0.493
EF (%)	58.00 [56.00, 60.00]	58.00 [57.00, 60.00]	58.00 [56.00, 60.00]	0.458
Insulin (%)	40 (27.2)	26 (24.1)	14 (35.9)	0.207
Metformin (%)	60 (40.8)	52 (48.1)	8 (20.5)	0.003**
TZD (%)	1 (0.7)	1 (0.9)	0 (0.0)	1
SU (%)	27 (18.4)	18 (16.7)	9 (23.1)	0.469
Glinide (%)	6 (4.1)	1 (0.9)	5 (12.8)	0.005**
Glucosidase inhibitor (%)	57 (38.8)	38 (35.2)	19 (48.7)	0.179
GLP1 (%)	147 (100.0)	108 (100.0)	39 (100.0)	NA
DPP-4i (%)	32 (21.8)	22 (20.4)	10 (25.6)	0.503
SGLT2i (%)	13 (8.8)	8 (7.4)	5 (12.8)	0.331
Anticoagulant (%)	6 (4.1)	5 (4.6)	1 (2.6)	1
Antiplatelet (%)	67 (45.6)	48 (44.4)	19 (48.7)	0.709
B-blocker (%)	47 (32.0)	35 (32.4)	12 (30.8)	1
Statins (%)	99 (67.3)	71 (65.7)	28 (71.8)	0.554
Antiarrhythmias (%)	7 (4.8)	5 (4.6)	2 (5.1)	1
ACEI/ARB (%)	65 (44.2)	51 (47.2)	14 (35.9)	0.261
Sacubatrovalsartan (%)	4 (2.7)	3 (2.8)	1 (2.6)	1
Aldactone (%)	15 (10.2)	13 (12.0)	2 (5.1)	0.355
Digoxin (%)	147 (100.0)	108 (100.0)	39 (100.0)	NA
CCB (%)	2 (1.4)	2 (1.9)	0 (0.0)	1
Furosemide (%)	14 (9.5)	9 (8.3)	5 (12.8)	0.524

**AF:** atrial fibrillation; **ACEI**: angiotensin converting enzyme inhibitor; **ARB**: angiotensin receptor blocker; **CCB**: calcium channel blockers; **CKD**: chronic kidney disease; **Cr**: creatinine; **DPP-4i**: dipeptidyl peptidase 4 inhibitor; **GLP1**: glucagon-like peptide-1; **HF:** heart failure; **SGLT2i**: sodium-dependent glucose transporters 2 inhibitor; **SU**: sulfonylurea; **TZD**: thiazolidinediones.

**Table 3:** Multivariable analysis of new-onset AF.

	HR (Model 1)	p	HR (Model 2)	p	HR (Model 3)	p
Metformin	0.37 (0.16–0.82)	0.014*	0.4 (0.18–0.89)	0.0258*	0.36 (0.14–0.91)	0.0311*

Adjusted for age, sex, BMI, drinking, smoking, LAD, Cr, complications, and drugs.

**HR**: hazard ratio.

### 3.2 Metformin did not affect AF burden in type 2 diabetes patients with paroxysmal AF

The clinical characteristics of type 2 diabetes patients with paroxysmal AF are shown in Table 4. No statistically significant difference was observed in the baseline data between the metformin and non-metformin groups. During follow-up, the AF burden was not significantly different between the two groups.

**Table 4:** Clinical characteristics of DM patients with paroxysmal atrial fibrillation.

	Overall (n = 80)	Non-metformin (n = 55)	Metformin (n = 25)	p
Female sex (%)	33 (41.2)	24 (43.6)	9 (36.0)	0.627
Age (year)	75.00 [67.75, 82.25]	76.00 [68.00, 83.00]	73.00 [66.00, 79.00]	0.333
BMI (kg/m^2^)	23.95 [21.95, 26.79]	23.95 [21.97, 26.23]	23.76 [21.62, 27.43]	0.53
Systolic pressure (mmHg)	126.00 [122.00, 135.00]	128.00 [121.50, 135.00]	126.00 [124.00, 134.00]	0.735
Dystolic pressure (mmHg)	68.81 (8.63)	67.80 (9.05)	71.04 (7.31)	0.12
Hypertension (%)	60 (75.0)	40 (72.7)	20 (80.0)	0.585
Hypercholesterol (%)	14 (17.5)	10 (18.2)	4 (16.0)	1
CVD (%)	26 (32.5)	18 (32.7)	8 (32.0)	1
MI (%)	9 (11.2)	6 (10.9)	3 (12.0)	1
HF (%)	18 (22.5)	12 (21.8)	6 (24.0)	1
Stroke (%)	11 (13.8)	4 (7.3)	7 (28.0)	0.03*
CKD (%)	22 (27.5)	16 (29.1)	6 (24.0)	0.789
Drinking (%)	8 (10.0)	5 (9.1)	3 (12.0)	0.7
Smoking (%)	14 (17.5)	11 (20.0)	3 (12.0)	0.531
Cr (μmol/L)	78.00 [62.00, 93.75]	81.50 [62.25, 101.00]	74.50 [60.50, 84.97]	0.262
CRP (mg/L)	3.20 [2.30, 4.95]	3.10 [2.30, 4.90]	3.65 [2.25, 5.50]	0.811
HbA1c (%)	6.80 [6.30, 7.65]	6.75 [6.30, 7.48]	6.90 [6.30, 7.95]	0.613
LAD (cm)	4.39 (0.51)	4.41 (0.56)	4.36 (0.40)	0.715
EF (%)	58.00 [55.00, 59.50]	57.00 [54.50, 59.00]	58.50 [55.00, 60.00]	0.252
Insulin (%)	17 (21.2)	14 (25.5)	3 (12.0)	0.242
TZD (%)	80 (100.0)	55 (100.0)	25 (100.0)	NA
SU (%)	19 (23.8)	11 (20.0)	8 (32.0)	0.267
Glinide (%)	3 (3.8)	3 (5.5)	0 (0.0)	0.548
Glucosidase inhibitor (%)	33 (41.2)	24 (43.6)	9 (36.0)	0.627
GLP1 (%)	80 (100.0)	55 (100.0)	25 (100.0)	NA
DPP-4i (%)	10 (12.5)	9 (16.4)	1 (4.0)	0.16
SGLT2i (%)	16 (20.0)	13 (23.6)	3 (12.0)	0.366
Anticoagulant (%)	48 (60.0)	30 (54.5)	18 (72.0)	0.218
Antiplatelet (%)	15 (18.8)	10 (18.2)	5 (20.0)	1
B-blocker (%)	44 (55.0)	30 (54.5)	14 (56.0)	1
Statins (%)	51 (63.7)	35 (63.6)	16 (64.0)	1
Anti-arrhythmia (%)	27 (33.8)	18 (32.7)	9 (36.0)	0.803
ACEI/ARB (%)	37 (46.2)	22 (40.0)	15 (60.0)	0.146
Sacubatrovalsartan (%)	7 (8.8)	7 (12.7)	0 (0.0)	0.092
Aldactone (%)	13 (16.2)	11 (20.0)	2 (8.0)	0.212
Digoxin (%)	1 (1.2)	1 (1.8)	0 (0.0)	1
CCB (%)	80 (100.0)	55 (100.0)	25 (100.0)	NA
Furosemide (%)	12 (15.0)	10 (18.2)	2 (8.0)	0.323
AF burden (%)	1.00 [0.00, 4.25]	1.00 [0.00, 3.85]	1.00 [0.00, 4.40]	0.817

**AF:** atrial fibrillation; **ACEI**: angiotensin converting enzyme inhibitor; **ARB**: angiotensin receptor blocker; **CCB**: calcium channel blockers; **CKD**: chronic kidney disease; **Cr**: creatinine; **DM:** diabetes mellitus; **DPP-4i**: dipeptidyl peptidase 4 inhibitor; **GLP1**: glucagon-like peptide-1; **HF:** heart failure; **SGLT2i**: sodium-dependent glucose transporters 2 inhibitor; **SU**: sulfonylurea; **TZD**: thiazolidinediones.

### 3.3 Metformin decreased the incidence of MACEs in diabetes patients

To investigate the effect of metformin use on MACEs in type 2 diabetes patients, we used multivariable Cox hazard models to compare the incidence of MACEs in the total, metformin, and non-metformin groups. We found a lower proportion of MACEs when adjusted for metformin, age, sex, BMI, alcohol consumption, smoking status, CVD, MI, HF, stroke, and EF in both the total (HR: 0.28; 95 % CI: 0.1–0.82; p = 0.0202*) and non-AF groups (HR: 0.19; 95 % CI: 0.05–0.79; p = 0.0223*). No significant difference was noted in the AF group (HR: 0.31; 95 % CI: 0.02–4.41; p = 0.3879) (Table 5).

**Table 5:** Risk of metformin use on MACEs.

	HR (Model 1)	p	HR (Model 2)	p	HR (Model 3)	p
MACE						
Total	0.32 (0.12–0.84)	0.0209*	0.26 (0.1–0.72)	0.0096**	0.28 (0.1–0.82)	0.0202*
non-AF	0.25 (0.07–0.87)	0.0295*	0.16 (0.04–0.63)	0.0086**	0.19 (0.05–0.79)	0.0223*
AF	0.38 (0.07–2)	0.2534	0.54 (0.08–3.66)	0.5291	0.31 (0.02–4.41)	0.3879

Model 1: Adjusted for metformin, age and gender.

Model 2: Adjusted for metformin use, age, sex, BMI, alcohol consumption, and smoking.

Model 3: Adjusted for metformin use, age, sex, BMI, alcohol consumption, smoking, CVD, MI, HF, Stroke and EF.

**AF:** atrial fibrillation; **HF:** heart failure; **HR**: hazard ratio; **MACEs**: major adverse cardiovascular events.

## 4 Discussion

The major findings of this retrospective observational study were as follows. 1) Metformin decreased the risk of new-onset AF in type 2 diabetes patients with pacemakers. 2) Metformin did not affect the AF burden in type 2 diabetes patients with pacemakers, including those with paroxysmal AF. 3) Metformin decreased the rate of MACEs in type 2 diabetes patients without AF compared to those with paroxysmal AF.

Metformin is the most widely used antidiabetic drug for type 2 DM and exerts its antidiabetic effect mainly by inhibiting hepatic gluconeogenesis. In addition to its antihyperglycemic effect, metformin has various cardiovascular benefits, including a reduction in HF, MI, stroke, cardiovascular death, and all-cause mortality [12]. According to a literature review [13], metformin is beneficial for heart metabolism, function, and structure. Many clinical trials have demonstrated that metformin can reduce HF mortality, lower the risk of new-onset HF, and reduce admissions for HF. These benefits may be associated with improved myocardial energy metabolism and efficiency, anti-oxidative and anti-inflammatory, reduction in epicardial adipose tissue [14], among others. HF and AF share a common pathological basis and interact through mechanisms such as cardiac structural remodelling, neurohormonal system activation, and heart rate-related left ventricular injury, leading to disease progression [15,16]. However, the exact role of metformin in arrhythmias remains unclear.

A retrospective observational study involving type 2 DM patients with AF demonstrated that metformin treatment is associated with a lower risk of recurrent atrial arrhythmias after catheter ablation [17]. However, prior use of metformin therapy in diabetes patients undergoing cardiac surgery was not associated with a decreased risk of postoperative AF [18]. Our study revealed a significant protective role of metformin against AF in type 2 diabetes patients with pacemakers.

A retrospective study observed that for every 5 kg of absolute weight loss, the risk of incident AF was reduced by 12 % in obese patients [19]. Since hyperglycaemia and obesity both increase susceptibility to AF, the prevention of AF in type 2 diabetes patients by metformin might be attributed to its antihyperglycemic and weight loss effects, providing straightforward explanations [20]. However, changes in blood glucose levels and BMI were not assessed in the present study. Therefore, these indices should be incorporated into future studies.

AMP-activated protein kinase (AMPK) is an essential protein in chronic metabolic diseases, such as type 2 diabetes, obesity, and cardiovascular disease [21]. Many animal experiments have proposed that metformin prevents AF by activating the AMPK pathway, given its role as an AMPK activator. Metformin or aspirin (an AMPK activator) prevents AF in a cardiac-specific liver kinase B1 knockout mouse model of AF [22]. Furthermore, metformin alleviates the vulnerability of AF, attenuates the downregulation of gap junctions under pacing conditions via the AMPK pathway, and decreases P-Src levels [23]. In a canine model of AF, metformin improved lipid metabolism and reversed the Warburg effect via AMPK activation [24]. A study also demonstrated that metformin reduced AF vulnerability and atrial fibrosis in a canine model by inhibiting ROS/NF-κB activation and upregulated PPARγ/APN expression [25].

AF burden represents the percentage of time an individual spends in AF during a monitoring period [26]. Stratification of the AF burden could objectively measure the effectiveness of AF treatment. A lower AF burden is associated with a decreased incidence of stroke [27], HF [28,29], and death [30]. Our findings suggest that metformin does not relieve the AF burden in type 2 diabetes patients with paroxysmal AF. These findings may also explain why metformin reduced MACEs in type 2 diabetes patients without AF rather than in those with paroxysmal AF.

The sodium-dependent glucose transporter 2 inhibitor (SGLT2i) is highly anticipated for its potential to improve HF, offering cardiovascular benefits beyond just lowering blood sugar levels. However, whether SGLT2i use prevents AF remains unclear. A meta-analysis of randomised controlled trials evaluating the effects of SGLT2i on AF in HF patients found that SGLT2i may have no preventive effect on AF [31]. Conversely, the Canagliflozin and Renal Events in Diabetes with Stabilised Nephropathy Clinical Evaluation trial and a meta-analysis of large cardiovascular outcome studies of SGLT2i in type 2 DM showed that SGLT2i may prevent AF, indicating its antiarrhythmic role [32]. In the present study, we found that SGLT2i increased the risk of AF. This can be attributed to the following reasons. First, the enrolled patients were equipped with pacemakers, and new-onset AF was detected through pacemaker testing, which differs from that in existing research. Our study is more accurate than other studies in detecting new-onset AF. In addition, the small sample size of SGLT2i users (13 of 147) limited the generalisability of our conclusion in terms of SGLT2i. Second, HF patients with a reduced ejection fraction and a dual-chamber pacemaker were analysed. These patients may benefit from SGLT2i use; however, dual-chamber pacemakers may exacerbate AF. However, our analysis did not consider this factor, which may have affected our conclusions. A multicentre prospective study is warranted to confirm the effect of SGLT2i on AF.

Our study had several strengths. Previous studies have used a 12-lead electrocardiogram or dynamic electrocardiogram to diagnose AF, consequently missing asymptomatic patients with paroxysmal AF and leading to inaccurate morbidity. To our knowledge, this is the first study to use a pacemaker to detect new-onset AF in type 2 diabetes patients. Additionally, the pacemaker provided data on the AF burden.

This study also had certain limitations. This was a retrospective observational study, and we could not establish a causal relationship between metformin use and the outcomes observed. In addition, the relatively small sample size may have weakened the statistical ability to detect subtle differences. A large-scale prospective study is required to confirm the generalisability of our findings. Medication changes were self-reported by the patients and were susceptible to recall bias. Finally, although we included patients with pacemakers and excluded those with CRT/D implants for the analysis, it is unclear whether these findings can be applied to all type 2 diabetes patients.

## 5 Conclusion

Our findings indicate that metformin reduced the probability of new-onset AF in diabetes patients with pacemakers, rather than affecting AF burden. Furthermore, metformin therapy decreased MACEs in diabetes patients without AF. Our findings highlight the importance of metformin therapy in diabetes patients, particularly those without AF.

## Ethics statement

This study was reviewed and approved by the Ethics Committee of Nanjing Drum Tower Hospital (approval number: 2022-208). All the participants provided informed consent to participate in the study.

## Funding

None.

## Conflict of interest

The authors declare that they have no known competing financial interests or personal relationships that could have appeared to influence the work reported in this paper.

## Data availability statement

The data associated with this study have not been deposited in a publicly available repository and will be made available on request.

## CRediT authorship contribution statement

**Chongxia Zhong:** Writing – original draft, Formal analysis, Data curation. **Jian Bai:** Formal analysis. **Xinhong Qu:** Writing – original draft. **Yihai Liu:** Data curation. **Zhu Li:** Data curation. **Han Hao:** Data curation. **Shiyang Qiao:** Data curation. **Zhe Zhang:** Data curation. **Xiaoying Xu:** Data curation. **Jiayi Si:** Data curation. **Wei Xu:** Methodology. **Biao Xu:** Methodology, Conceptualization. **Lina Kang:** Supervision, Methodology, Conceptualization.

## Declaration of competing interest

The authors declare that they have no known competing financial interests or personal relationships that could have appeared to influence the work reported in this paper.
